# How scientists can reduce their carbon footprint

**DOI:** 10.7554/eLife.15928

**Published:** 2016-03-31

**Authors:** Jeremy Nathans, Peter Sterling

**Affiliations:** 1 Howard Hughes Medical Institute, Johns Hopkins University School of Medicine, Baltimore, United States; 2 University of Pennsylvania, Philadelphia, United States

**Keywords:** Point of view, Climate change, Scientific meetings

## Abstract

Cutting down on long-distance air travel is the best way to reduce the emission of greenhouse gases by the scientific community.

Anthropogenic climate change is the single greatest challenge facing our planet and our species. What was once “an inconvenient truth” has become an imminent global emergency that will lead to rising seas, the extinction of species and, very likely, large-scale social instability. Thus far, however, government actions have had only modest effects on reducing greenhouse gas emissions. It is clear that individuals and communities need to take independent action.

Here we propose a set of relatively simple actions that could be implemented rapidly by the world’s scientists. While these actions represent only one small step in the right direction, we believe that if the scientific community leads by example, other communities will follow. The basic idea is to dramatically reduce long-distance air travel.

Although aircraft are becoming more fuel efficient, the global aviation industry has an enormous carbon footprint: the combustion of ~5 million barrels of jet fuel per day (Index Mundi, 2016) results in the daily release of ~2.4 million metric tons of carbon dioxide. Moreover, commercial aviation is the fastest growing source of greenhouse gas emissions, and it is projected that the world’s commercial fleets will triple their output of carbon dioxide by 2050 (Climate Law Institute, 2015).

## The carbon footprint of a scientist

For economy class travel on a commercial airplane, ~1 kg of carbon dioxide is emitted per passenger per 10 kilometers: this means that a flight from the east coast of the United States to central Europe and back (a distance of about 13,000 km) produces 1.3 metric tons of carbon dioxide per passenger. For comparison, a daily round-trip commute of 25 kilometers in a fuel-efficient car produces 1.5 metric tons of carbon dioxide per year. Thus, flying across the Atlantic and back generates roughly the same carbon footprint as a typical year of commuting by car.

To put these numbers in a global perspective, the mean carbon dioxide emission per person per year in the United States is 17 metric tons. Comparable figures for other countries include 9.3 for Japan, 6.7 for China, 5.2 for France, and 1.7 for India (World Bank, 2016). These carbon footprints reflect the total economic activity of a given country, so the carbon dioxide emissions that are under the control of an individual citizen are substantially smaller. Moreover, the per capita carbon footprint is expected to rise substantially in many countries in the developing world over the next 50 years, driven by a broad increase in the standard of living, so citizens in the developed world need to do all they can to start reducing carbon dioxide emissions now.

How does the carbon footprint of air travel compare to the carbon footprint of a typical life sciences laboratory? If we ignore the energy consumed in the heating and cooling of laboratory buildings – values that vary widely depending on locale – we find that most of the carbon footprint is due to refrigerators, freezers and other large items of equipment. For example, it takes ~18 kW-hours per day to operate a freezer at -80°C, which translates into 4 metric tons of carbon dioxide per year (based on the US average of 1.34 pounds of carbon dioxide per kW-hour; Union of Concerned Scientists, 2012). When lighting, cold rooms and smaller items of laboratory equipment (such as computers, spectrophotometers and incubators) are included, we estimate that the equipment in a typical life sciences laboratory of 7–10 people would likely generate more than 20 metric tons of carbon dioxide per year.

It is difficult to envision how life scientists could significantly reduce the carbon footprint associated with operating their laboratories since virtually all of the energy is used to run essential equipment. Thus, long-distance air travel would appear to be the largest source of work-related carbon dioxide emission that the scientific community could easily reduce.

To illustrate the magnitude of the scientific community’s travel-related carbon footprint, consider the annual meeting of the Society for Neuroscience (SFN), which attracts ~30,000 attendees to the United States from across the globe. By analyzing the cities of origin of attendees at the 2014 SFN meeting in Washington, DC, we estimate the mean round-trip distance traveled per person at ~7,500 kilometers, which gives the meeting a carbon footprint of 22,000 metric tons, roughly equivalent to the annual carbon footprint of 1000 medium-sized laboratories.

## A modest proposal

As a first step in reducing the carbon footprint of the scientific community we propose that all large scientific societies with an annual meeting cut back to one large meeting every two years. To partially compensate, we envision a series of smaller local meetings in alternate years that would bring together hundreds or thousands, rather than tens of thousands, of participants. The proposed change would roughly halve the carbon footprints associated with the largest meetings. We have formally proposed this idea to Dr. Hollis Cline, President of the Society for Neuroscience, and she has agreed to put it before the Society’s council. As a practical matter, any proposed changes in the scheduling of large national or international meetings would likely occur on a time scale of at least 5–10 years because societies have to book convention centers many years in advance. This is all the more reason to start the process sooner rather than later.

We further propose that seminars, grant review panels, interviews and other travel-intensive activities be examined with an eye toward reducing long-distance travel. For each of these activities, we should ask whether there is an equivalent seminar speaker or committee member who could travel 300 km instead of 3,000 km. For activities that do not require a face-to-face meeting, such as grant reviews, we should ask whether remote video-conferencing or a conference call could accomplish the same or nearly the same end. Scientists who do not live and work close to major scientific hubs may fear that a reduction in long-distance travel will lead to scientific isolation. Solutions to this challenge might include traveling with an itinerary that includes multiple clustered destinations and making greater use of electronic communication technology.

If scientists do not lead by example, then who else will?

We appreciate that face-to-face meetings play an important role in the life of a scientific field. However, the cost-benefit balance has shifted: climate-change costs are long-term and they are going to be staggeringly disruptive. This new reality calls for greater flexibility and greater creativity in how we conduct our business. Fortunately, we are more connected now than ever before, thanks to today’s technology, so we can offset a reduction in face-to-face meetings by making greater use of electronic communication. For example, when one of us (JN) was invited to lecture in Hyderabad, India, the lecture, together with a question-and-answer session, was delivered in real-time via a remote video-link from a studio at Johns Hopkins University in the US. With video screens simultaneously showing the speaker, the audience and the PowerPoint presentation, the experience was remarkably similar to a live presentation. The entire event took the speaker less time than he would have spent driving to the airport and waiting to board the plane to India. It also vacated an airplane seat that would have generated 2.6 metric tons of carbon dioxide.

Reductions in long distance travel by the scientific community, if adopted on a global scale, could reduce carbon dioxide emissions by many hundreds of thousands of metric tons annually. Moreover, a demonstration by the scientific community that it is taking concrete action would be an important step in convincing the general public to consider similar actions. Conversely, if scientists – arguably the most highly educated, informed and intellectually rigorous members of society – do not lead by example, then who else will lead?

In summary, the changes that we propose are not small, but they are modest compared to the changes that will soon be forced upon humanity by anthropogenic climate change.

**Figure 1:**
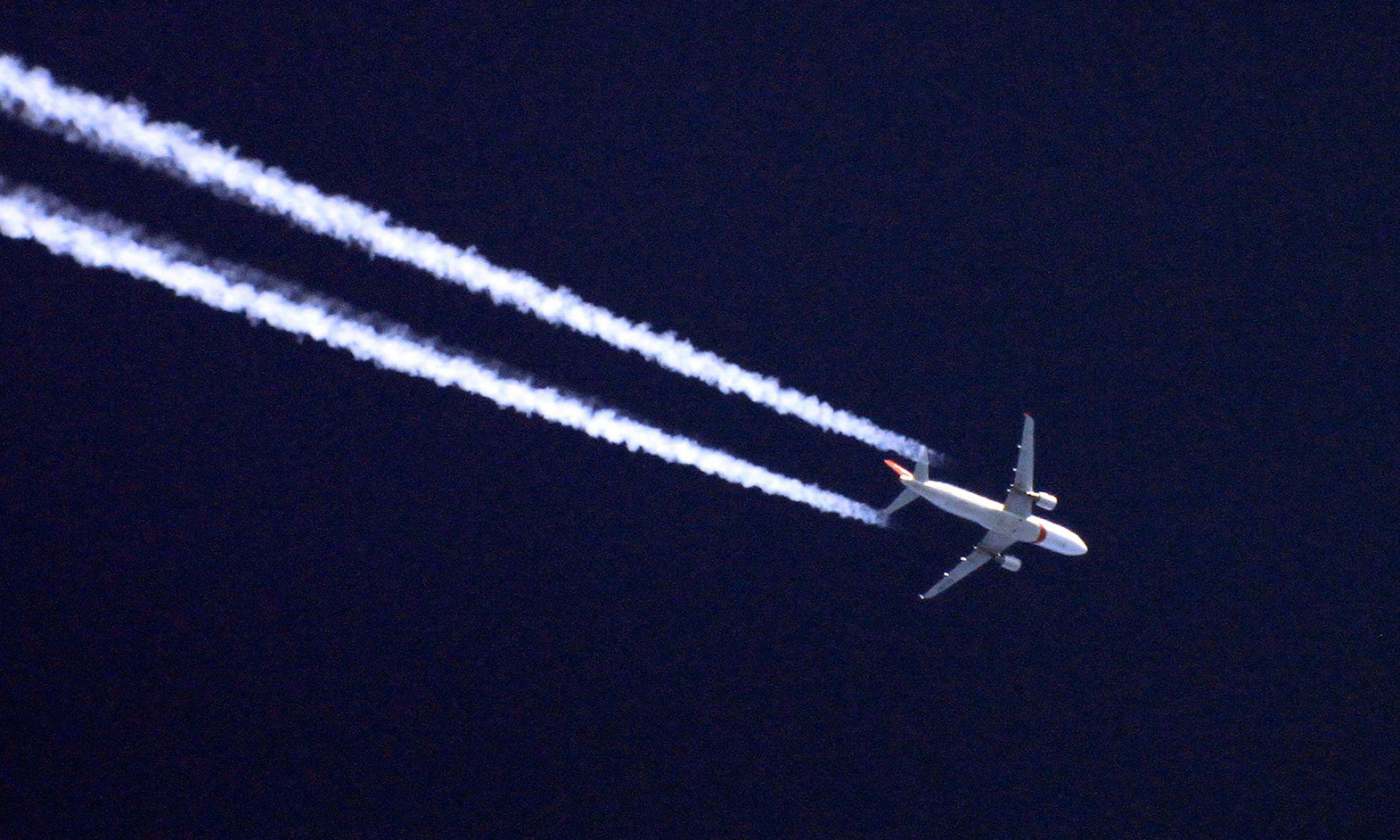
Scientists can reduce their carbon footprint by reducing long-distance air travel, and scientific societies could help by reducing the frequency of large international meetings. Photograph: Sebastien Lebrigand/CC-BY-SA-2.0
